# Caracterización molecular del dominio de la hélice del gen *k13* de *Plasmodium falciparum* en muestras de comunidades nativas de Condorcanqui, Amazonas, Perú

**DOI:** 10.7705/biomedica.6849

**Published:** 2023-09-30

**Authors:** Julio Sandoval-Bances, Milagros Saavedra-Samillán, Sonia Huyhua-Gutiérrez, Luis M. Rojas, Sonia Tejada-Muñoz, Rafael Tapia-Limonchi, Stella M. Chenet

**Affiliations:** 1 Instituto de Enfermedades Tropicales, Universidad Nacional Toribio Rodríguez de Mendoza de Amazonas, Triunfo, Chachapoyas, Perú Universidad Nacional Toribio Rodríguez de Mendoza de Amazonas Instituto de Enfermedades Tropicales Universidad Nacional Toribio Rodríguez de Mendoza de Amazonas Triunfo, Chachapoyas Peru; 2 Facultad de Ciencias de la Salud, Universidad Nacional Toribio Rodríguez de Mendoza de Amazonas, Triunfo, Chachapoyas, Perú Universidad Nacional Toribio Rodríguez de Mendoza de Amazonas Facultad de Ciencias de la Salud Universidad Nacional Toribio Rodríguez de Mendoza de Amazonas Triunfo, Chachapoyas Peru; 3 Dirección Regional de Salud de Amazonas, Chachapoyas, Perú Dirección Regional de Salud de Amazonas Chachapoyas Perú; 4 Instituto de Investigaciones en Ciencias Biomédicas, Universidad Ricardo Palma, Lima, Perú Universidad Ricardo Palma Instituto de Investigaciones en Ciencias Biomédicas Universidad Ricardo Palma Lima Peru

**Keywords:** Plasmodium falciparum, malaria, resistencia a medicamentos, ecosistema amazónico, Perú, Plasmodium falciparum, malaria, drug resistance, Amazonian ecosystem, Perú

## Abstract

**Introducción.:**

La resistencia de *Plasmodium falciparum* a diferentes fármacos antipalúdicos es un obstáculo para eliminar la enfermedad. El genotipo resistente de *P. falciparum* a la artemisinina puede evaluarse examinando los polimorfismos en el dominio de la hélice del gen *Pfk13*. La Organización Mundial de la Salud recomienda utilizar estas mutaciones como marcadores moleculares para detectar la resistencia a la artemisinina en países donde la malaria por *P. falciparum* es endémica.

**Objetivo.:**

Identificar mutaciones relacionadas con la resistencia a artemisinina presentes en el dominio de la hélice del gen *k13* de *P. falciparum*.

**Materiales y métodos.:**

Mediante la detección pasiva de casos, se recolectaron 51 muestras positivas por microscopía para *Plasmodium*, provenientes de seis comunidades del distrito de Río Santiago en Condorcanqui, Amazonas. Se realizó la confirmación molecular de la especie mediante PCR en tiempo real y el dominio de la hélice del gen *Pfk13* se amplificó y secuenció por electroforesis capilar. Las secuencias obtenidas se compararon con la cepa de referencia 3D7 de fenotipo silvestre.

**Resultados.:**

Se confirmó un total de 51 muestras positivas para *P. falciparum,* provenientes de las comunidades de Ayambis, Chapiza, Palometa, Muchinguis, Alianza Progreso y Caterpiza. Después del alineamiento de las secuencias de ADN, se determinó que las muestras no presentaron mutaciones asociadas con resistencia en el gen *K13*.

**Discusión.:**

Los resultados obtenidos son coherentes con estudios similares realizados en otros países de Sudamérica, incluyendo Perú. Estos datos proporcionan una línea base para la vigilancia molecular de resistencia a artemisinina en la región Amazonas y refuerzan la eficacia de la terapia combinada con artemisinina en esta área.

## Abstract (en)

**Introduction.:**

Resistance of *Plasmodium falciparum* to different antimalarial drugs is an obstacle to disease elimination. The artemisinin-resistant genotype of *P. falciparum* can be assessed by examining polymorphisms in the helix domain of the *Pfk13* gene. The World Health Organization recommends these mutations as molecular markers to detect artemisinin-resistant in countries where *P. falciparum* malaria is endemic.

**Objective.:**

To identify artemisinin resistance-related mutations present in the helix domain of the *P. falciparum k13* gene.

**Materials and methods.:**

We collected a total of 51 samples through passive case detection, positive for *Plasmodium* by microscopy, from six communities in the district of Río Santiago in Condorcanqui, Amazonas. Molecular species confirmation was performed by real-time PCR and *Pfk13* helix domain was amplified and sequenced by capillary electrophoresis. The obtained sequences were compared with the wild type 3D7 reference strain.

**Results.:**

A total of 51 positive samples were confirmed for *P. falciparum* from the communities of Ayambis, Chapiza, Palometa, Muchinguis, Alianza Progreso and Caterpiza. DNA sequences alignment showed the absence of resistance-associated mutations in the *k13* gene of the collected samples.

**Discussion.:**

The obtained results are consistent with similar studies conducted in other South American countries, including Perú, so these data provide a baseline for artemisinin- resistance molecular surveillance in the Amazon region and reinforce the efficacy of artemisinin-based combination therapy in this area.

La malaria es una enfermedad parasitaria transmitida por mosquitos hembra del género *Anopheles*. La mayoría de los casos de malaria complicada y muertes reportadas, principalmente en niños menores de cinco años, son causados por la especie *Plasmodium falciparum*^1^.

En Latinoamérica, el tratamiento actual para la infección por *P. falciparum* es la terapia combinada basada en artemisinina (*Artemisininbased combination therapy*)^2^. Estas terapias combinan un derivado de la artemisinina y un fármaco asociado, como lumefantrina, mefloquina, amodiaquina, piperaquina o pironaridina. Estos tratamientos han contribuido a reducir la mortalidad y la morbilidad relacionadas con el paludismo ^3^. Sin embargo, históricamente *P. falciparum* ha presentado resistencia a casi todos los antipalúdicos disponibles, por lo cual se requiere conocer adecuadamente la eficacia de los medicamentos antipalúdicos ^2^.

La resistencia de *P. falciparum* a la artemisinina, definida como un retraso en la eliminación de los parásitos del torrente sanguíneo, fue reportada por primera vez en la frontera entre Tailandia y Camboya, y en 10 años, las cepas resistentes a artemisinina se extendieron rápidamente en toda la cuenca del río Mekong, lo que resultó en grandes desafíos para la prevención y el tratamiento de la malaria ^4^. Los estudios de asociación genómica vincularon un locus en el cromosoma 13 de *P. falciparum* con la resistencia a artemisinina, específicamente, el dominio de la hélice del gen que codifica para la proteína Kelch - *Pfk13*^5^. Las mutaciones relacionadas con la resistencia a artemisinina en el dominio de la hélice del gen *Pfk13* son ocho (P441L, F446I, S449A, N458Y, P553L, V568G, P574L y L675V) asociadas con retraso en la eliminación del parásito en sangre, y cinco (Y493H, R539T, I543T, R561H y C580Y) relacionadas con resistencia *in vitro*, *in vivo* o en ambos ^1^. La Organización Mundial de la Salud (OMS) recomienda utilizar estas mutaciones para detectar la presencia de resistencia a la artemisinina en países donde la malaria por *P. falciparum* es endémica. Asimismo, sugiere realizar la vigilancia molecular de forma regular en lugares donde no se pueden llevar a cabo estudios clínicos por otras causas, como su aislamiento geográfico ^1^.

Las mutaciones reportadas del gen *Pfk13* varían en diferentes regiones. Por ejemplo, en Camboya, Vietnam y Laos, la mutación C580Y es dominante ya que tiene la prevalencia más alta, con un 50 %; en la frontera entre Tailandia y Myanmar, se ha reportado resistencia a la artemisinina con un mayor número de mutaciones C580Y y menor número de mutaciones N458Y ^6^; y en África, la mutación más común y específica es la A578S ^7^. En Sudamérica, se han encontrado parásitos mutantes C580Y, reportados en Guyana ^8^.

Las investigaciones han revelado que la mutación C580Y en Guyana no se propagó desde el sudeste asiático, sino que emergió en el Escudo Guyanés de forma independiente ^3^^,^^8^. Es por ello que el sudeste asiático y el Escudo Guyanés son considerados puntos críticos para la resistencia a los medicamentos antipalúdicos, lo que se ha evidenciado anteriormente con la resistencia a cloroquina y sulfadoxina-pirimetamina ^9^.

La artemisinina y sus derivados se asocian con otros medicamentos antipalúdicos para retrasar el desarrollo de resistencia. Por ello, existe la preocupación de que la reducción de la sensibilidad a la artemisinina conduzca al desarrollo de resistencia de los parásitos a los medicamentos asociados que juegan un papel crucial en la eficacia general de las terapias combinadas de artemisinina ^3^^,^^8^.

Actualmente, los tratamientos combinados son eficaces. Sin embargo, la aparición y expansión de parásitos resistentes a la artemisinina podrían afectar su eficacia general ^8^, sobre todo en áreas de poca transmisión, donde la eliminación de la malaria está en curso ^2^.

En Perú, la política de tratamiento contra *P. falciparum* basado en combinaciones con artemisinina se implementó en el 2001 ^10^. De acuerdo con la norma técnica vigente, el tratamiento de la malaria no complicada por *P. falciparum* consiste en una combinación de mefloquina (12,5 mg/ kg/día durante dos días) más artesunato (4 mg/kg/día durante tres días) y primaquina (10 mg/kg/día para adulto y 0,75 mg/kg/día para niños durante un día) ^11^. No obstante, en los últimos años no se han realizados estudios clínicos para evaluar la eficacia del tratamiento combinado de mefloquina y artesunato en pacientes con *P. falciparum* en el Amazonas. Este trabajo es el primer reporte sobre vigilancia molecular de la resistencia a artemisinina en el departamento de Amazonas y será útil para orientar los esfuerzos para eliminar la malaria en Perú.

## Materiales y métodos

### Obtención de muestras y sitio de estudio

**Figura 1:**
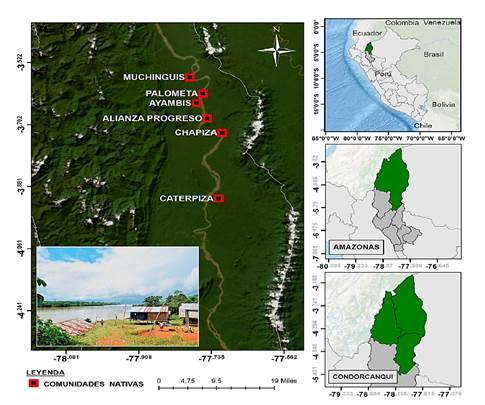
Mapa de los sitios de recolección en el distrito de Río Santiago, Provincia de Condorcanqui, Amazonas, Perú. Se muestran las comunidades nativas ubicadas a lo largo del río Santiago: Palometa, Ayambis, Chapiza, Alianza Progreso, Muchinguis y Caterpiza.

Las muestras, recolectadas en papel filtro siguiendo los procedimientos estándar de la OMS ^12^, fueron proporcionadas por la Dirección Regional de Salud, mediante la detección pasiva de casos durante el periodo 2019-2022. Se obtuvieron muestras positivas para *Plasmodium*, identificadas por microscopía, provenientes de seis comunidades del distrito de Río Santiago, provincia Condorcanqui, departamento del Amazonas (figura 1).

El sitio de estudio fue el distrito Río Santiago, donde se presenta un clima tropical húmedo con temperaturas que pueden alcanzar los 35 °C, precipitación media anual alrededor de 4.800 mm y humedad relativa superior al 90 %. La temporada de lluvias ocurre entre octubre y diciembre, pero podría durar hasta mayo ^13^.

Las muestras se recolectaron en las siguientes comunidades: en el 2019, en Ayambis (n = 5); en el 2020, en Ayambis (n=1) y Chapiza (n=13); en el 2021, en Alianza Progreso (n=7), Muchinguis (n=1) y Palometa (n=1); y en el 2022, en Alianza progreso (n=19), Chapiza (n=1) y Caterpiza (n=3).

### Extracción de material genético

La extracción de ADN se realizó utilizando el QIAamp DNA Mini Kit (Qiagen, EEUU), según las instrucciones del fabricante. El ADN obtenido se almacenó a -20 ^0^C.

### PCR en tiempo real para identificar Plasmodium falciparum

Para la detección de *P. falciparum* se empleó la técnica de PCR en tiempo real, según Rougemont *et al.*, 2004. La muestra se consideró positiva mediante la identificación del número del ciclo de amplificación (Ct) en el que se evidenció la emisión de fluorescencia del reportero. Si la señal fluorescente no se detectaba en 40 ciclos (Ct > 40), la muestra se consideraba negativa ^14^.

### *Genotipificación del dominio de la hélice del gen Pfk13 mediante PCR* anidada

El dominio de la hélice del gen *Pfk13* fue amplificado por PCR anidada en muestras con un ciclo de amplificación inferior a 40, de acuerdo con un protocolo previamente reportado ^8^. Para la primera PCR el volumen fue de 30 μl, usando 4 μl de ADN, solución tampón 1X, 2 mM de MgCl_2_, 0,2 mM de dNTP, 0,5 μM de cada cebador: *forward* (*K13Pf*_F1 5’GCAAATAGTATCTCGAAT-3’) y *reverse* (*K13Pf*_ R1 5CTGGGAACTAATAATAAAGAT-3’) y una unidad de *Platinum Taq polymerase* (Invitrogen); el volumen final se completó con agua libre de nucleasas. Para la segunda PCR, los cebadores utilizados fueron: K13 *Pf* _F2 5’GATAAACAAGGAAGAATATTCT-3‘ y K13 *Pf*_R2 5’CGGAATCTAATATGTTATGTTCA-3’. Se utilizaron las mismas concentraciones de la primera reacción. Las condiciones de corrida fueron las mismas que se indican en el protocolo previamente reportado ^8^.

La amplificación se realizó con el termociclador SimpliAmpTM Thermal Cycler (Applied Biosystems). Los productos de PCR se visualizaron en geles con agarosa al 2 % y con ayuda de un transiluminador.

### Purificación y secuenciación

Los productos de la PCR fueron purificados con el kit Exo-CIP™ (New England Biolabs) y secuenciados con el kit BigDye™ Terminator (Thermo Fisher Scientific). Los cebadores para la secuenciación fueron los mismos que los usados en la segunda PCR. Los productos amplificados se secuenciaron mediante electroforesis capilar en el equipo 3500 Genetic Analyzer (Applied Biosystems™).

### Análisis bioinformático

Los electroferogramas fueron analizados en el *software* Geneious Prime v.2022.2.1 (Biomatters Inc, Newark). La secuencia de referencia fue la 3D7 (PF3D7_1343700 K13) obtenida de la base de datos *del National Center for Biotechnology Information* (NCBI).

### Aspectos éticos

El estudio fue aprobado por el Comité de Ética de Investigación PI 15-2022 de la Facultad de Medicina “Manuel Huamán Guerrero” de la Universidad Ricardo Palma. Además, se obtuvo el consentimiento informado de cada participante o de sus asesores legales. Los métodos se llevaron a cabo de acuerdo con las directrices y normas pertinentes.

## Resultados

### Muestra y amplificación por PCR en tiempo real

Desde diciembre del 2019 hasta junio del 2022, se obtuvieron 51 muestras de seis comunidades nativas del distrito de Río Santiago. Las muestras se confirmaron por PCR en tiempo real, siendo todas positivas para *P. falciparum*, con ciclos de amplificación inferiores a 35.

### Amplificación por PCR anidada y secuenciación

En todas las muestras se amplific*ó* el dominio de la hélice del gen *Pfk13* por PCR anidada con un tamaño del producto de 700 pares de bases. Los 51 aislamientos se secuenciaron con éxito y se evaluaron los siguientes polimorfismos del gen *Pfk13*: P441L, F446I, N458Y, M476I, Y493H, R539T, I543T, P553L, R561H, V568G, P574L, C580Y y A675V. Todos los aislamientos de *P. falciparum* presentaron el alelo de tipo silvestre 3D7 en el dominio de la hélice del gen *Pfk13*, por lo que no se evidenciaron mutaciones en ninguna de las muestras analizadas (cuadro 1).

**Cuadro 1:** Ausencia de mutaciones en el dominio de la hélice del gen *K13*, en muestras de las comunidades nativas de Condorcanqui. Se exhibe el alelo de fenotipo silvestre 3D7 para todas las muestras.

				Comunidades				
Posición del codón	Aminoácido silvestre / mutado	Progreso n_2021=7_ n_2022=19_	Ayambis n_2019=5_ n_2020=1_	Caterpiza n_2022=3_	Chapiza n_2020=13_ n_2022=1_	Muchinguis n_2021=1_	Palometa n_2021=1_	Resultado
441	P / L	P	P	P	P	P	P	WT
446	F / I	F	F	F	F	F	F	WT
458	N / Y-I	N	N	N	N	N	N	WT
476	M / I-V	M	M	M	M	M	M	WT
493	Y / H	Y	Y	Y	Y	Y	Y	WT
539	R / T	R	R	R	R	R	R	WT
543	I / T	I	I	I	I	I	I	WT
553	P / L	P	P	P	P	P	P	WT
561	R / H	R	R	R	R	R	R	WT
568	V / G	V	V	V	V	V	V	WT
574	P / L	P	P	P	P	P	P	WT
580	C / Y	C	C	C	C	C	C	WT
675	A / V	A	A	A	A	A	A	WT

P: prolina, L: leucina, F: fenilalanina, I: isoleucina, N: asparagina, Y: tirosina, M: metionina, V: valina, H: histidina, R: arginina, T: treonina, G: glicina, C: cisteína, A: alanina, N: número de muestras, WT: Wild type (fenotipo silvestre)

## Discusión

La artemisinina es un fármaco antipalúdico que se usa principalmente para el tratamiento contra la malaria en países endémicos. Sin embargo, hay un problema emergente de resistencia a este antimalárico en África y Asia Tropical ^15^. En Sudamérica se ha reportado un genotipo resistente en el gen *Pfk13* (C580Y) en Guyana ^8^. Sin embargo, aún no se ha extendido a otros países de este continente ^2^.

La supuesta y relativamente baja exposición de la población brasileña de *P. falciparum* a la artemisinina antes del 2006, probablemente explique la ausencia de polimorfismos en el dominio de la hélice del gen *Pfk*13, incluso después de la introducción del tratamiento combinado como de primera línea en Brasil ^16^. En un estudio reciente en las cuencas del Amazonas brasileño, se analizaron los polimorfismos en el dominio de la hélice del gen *Pfk13* y se demostró que aún no existe evidencia de polimorfismos asociados con la resistencia a la artemisinina, por lo que continúa el uso del tratamiento combinado de artemisinina en la región de Brasil ^17^. Estos resultados son congruentes con los obtenidos en el presente estudio y permiten ampliar los conocimientos respecto a los parásitos circulantes de *P. falciparum* en Amazonas.

En otros estudios recientes con aislamientos brasileños, peruanos y colombianos, se ha evidenciado la ausencia de mutaciones en el *PfK13* y el tratamiento combinado de artemisinina parece ser efectivo en estas regiones ^18^^,^^19^. En Ecuador, se han realizado análisis génicos de las posiciones 476, 493, 539, 543, 580 para *Pfk13* y se han obtenido genotipos de tipo silvestre ^3^.

Mientras tanto, en Perú, en el último año se realizó un estudio de secuenciación muy multiplexado (AmpliSeq, Illumina) dirigido a 13 genes de resistencia a los antipalúdicos, incluyendo la longitud completa del gen *K13.* Como resultado de este estudio, se obtuvieron polimorfismos en frecuencias bajas, no asociadas con resistencia, lo que sugiere ausencia de resistencia a la artemisinina en Perú durante los últimos años ^20^.

En el presente estudio se determinó que no existen mutaciones en *Pfk13* asociadas con resistencia a la artemisinina en las comunidades de R**í**o Santiago, provincia de Condorcanqui. Sin embargo, aunque no se encontraron mutaciones de resistencia a artemisinina en las muestras estudiadas, no se puede descartar la propagación de otras mutaciones relacionadas con la resistencia a este fármaco. En varios estudios independientes sobre la adaptación a fármacos, han conseguido identificar diversos marcadores que están relacionados con la resistencia a la artemisinina en pruebas *in vitro.* Por esto, es probable que no exista un único “marcador universal” para la resistencia a la artemisinina ^21^. Asimismo, se han realizado investigaciones en la población mediante el análisis de genomas y transcriptomas, las cuales han permitido identificar múltiples regiones genómicas y genes con expresión diferencial; estos podrían estar relacionados con resistencia a los derivados de la artemisinina o a los fármacos asociados ^22^. La plasticidad genómica de *Plasmodium* puede propiciar el desarrollo relativamente rápido de resistencia a los medicamentos antipalúdicos, por lo que es importante implementar plataformas de vigilancia que permitan la detección temprana de resistencia en entornos endémicos ^19^. Además, es importante hacer seguimiento al paciente, pues el resultado de su incumplimiento del tratamiento puede confundirse con una falla terapéutica por resistencia al medicamento.

Actualmente, Perú cuenta con el “Plan hacia la eliminación de la malaria 2022-2030”, cuyo objetivo es implementar actividades para erradicar la malaria en el país mediante un plan con enfoque comunitario ^23^. Es por ello que entender y continuar con la vigilancia molecular de antimaláricos dirigidos contra *P. falciparum* es crucial para apoyar este programa y para la generación de estrategias de eliminación.

Finalmente, en el presente trabajo se muestra la importancia de los estudios moleculares como herramientas de vigilancia para ayudar al control y la eliminación de la malaria en el país. La detección temprana de la resistencia al tratamiento farmacológico actual utilizado para las infecciones por *P. falciparum*, es esencial para detener los brotes. Nuestros datos proporcionan una línea base para la caracterización del *Pfk13* y refuerzan la eficacia del tratamiento combinado con artemisinina.
